# Remifentanil Versus Fentanyl in Critically Ill Patients Requiring Mechanical Ventilation: A Single-Center Retrospective Cohort Study

**DOI:** 10.7759/cureus.87804

**Published:** 2025-07-13

**Authors:** Shinichiro Hasegawa, Hiromu Okano, Satoshi Jujo, Hiroshi Okamoto

**Affiliations:** 1 Department of Critical Care Medicine, St. Luke's International Hospital, Tokyo, JPN; 2 Department of Social Medical Sciences, Graduate School of Medicine, International University of Health and Welfare, Tokyo, JPN

**Keywords:** delirium, fentanyl, icu, mechanical ventilation, remifentanil

## Abstract

Background

Remifentanil, known for its ultra-short-acting properties and organ-independent metabolism, offers benefits over traditional opioids. Although approved for intensive care unit (ICU) use in Japan since 2022, its comparative effects against fentanyl in mechanically ventilated critically ill patients remain unclear. This study evaluated the effects of remifentanil versus fentanyl in this population.

Methods

This retrospective, single-center observational study included patients in ICUs requiring mechanical ventilation between April 2021 and May 2024 (excluding patients in postoperative status). Propensity score matching was performed to adjust for baseline differences. The primary outcome was mechanical ventilation duration. Secondary outcomes included delirium incidence and drug-associated costs. Statistical analyses were conducted using Mann-Whitney U and chi-square tests.

Results

Of 198 eligible patients, 31 were analyzed in each group following matching. The median (interquartile range) ventilation duration was 107.5 (45.6-196.8) and 124.1 (93.8-324.0) hours for remifentanil and fentanyl, respectively (median difference: -16.6 hours; 95% confidence interval: -147.0-26.2; P = 0.15), indicating statistical non-significance but a wide confidence interval that suggests the potential for a clinically meaningful difference. Delirium incidence was lower in the remifentanil group (77.4% vs. 96.4%; χ²(1) = 4.99, P = 0.03, φ = 0.29). Although daily analgesic costs were significantly higher for remifentanil than fentanyl ($29.5 vs. $13.9; P < 0.01), total sedation-analgesia costs were similar between the groups ($45.3 vs. $26.2; P = 0.13).

Conclusion

Although remifentanil did not significantly reduce ventilation duration compared to fentanyl, it was associated with a significantly lower delirium incidence, suggesting potential advantages in sedation.

## Introduction

Pain affects over 70% of patients in intensive care units (ICUs) [1]. Inadequate pain management can lead to adverse physiological effects and long-term complications, including chronic pain syndromes and post-traumatic stress disorder [2-4]. Fentanyl and morphine remain the primary opioids used in Japanese ICUs [5,6]. However, these opioids have limitations, particularly regarding accumulation in organ dysfunction and prolonged effects that may complicate ventilator weaning [6,7]. Following its successful use in anesthesia, remifentanil was approved for ICU sedation in Japan in August 2022 after a multicenter, randomized, double-blind phase 3 trial [7]. Regarding its effectiveness in ICU settings, Maddali et al. [8] reported reduced ventilation duration in post-cardiac surgery patients, while Liu et al. [9] reported decreased delirium incidence in a surgical ICU population.

A recent meta-analysis primarily focused on postoperative patients, and the evidence for remifentanil's efficacy in medically critically ill (non-surgical) patients remains limited. Although subgroup analysis from this meta-analysis suggested that remifentanil may shorten the duration of mechanical ventilation in non-surgical patients [10], this conclusion is based on a single randomized trial [11]. In that study, 19% of patients received both remifentanil and fentanyl, complicating the interpretation of results as a direct comparison between monotherapies. Moreover, the authors concluded that further research is needed to clarify the efficacy of remifentanil monotherapy compared to fentanyl in non-surgical critically ill populations. Compared to surgical patients, critically ill individuals, such as those with sepsis or respiratory failure, may have altered pharmacokinetics, increased organ dysfunction, and different sedation needs, which could influence both the efficacy and safety profile of opioids. These differences highlight the importance of evaluating remifentanil in non-surgical ICU populations specifically.

Given these limitations in the existing evidence, particularly regarding non-surgical critically ill populations, we aimed to compare the clinical effects of remifentanil versus fentanyl monotherapy in mechanically ventilated, critically ill patients, focusing on mechanical ventilation duration, incidence of delirium, ICU stay, and medication-related costs.

## Materials and methods

Study design, population, and setting

This single-center retrospective observational study was conducted at St. Luke's International Hospital in Tokyo, Japan, between April 2021 and May 2024. We followed the Strengthening the Reporting of Observational Studies in Epidemiology (STROBE) guidelines [12]. The study was approved by the Ethics Committee of St. Luke's International Hospital (approval no. 24-J012), and the requirement for informed consent was waived due to the retrospective nature of the study and the use of anonymized data.

The study population comprised critically ill adult patients (≥18 years) admitted to the ICU for medical conditions requiring mechanical ventilation. We excluded all surgical and postoperative patients to focus specifically on medical critical care cases. Additional exclusion criteria were: post-cardiac arrest [13], ICU readmissions, mechanical ventilation initiation >24 hours after ICU admission, ventilation duration <12 hours [11], unknown transfer status, unknown sedation dosage, concurrent use of remifentanil and fentanyl, tracheostomy at ICU admission, convulsive seizures [7], or incomplete height/weight data. These criteria were selected based on prior literature and clinical considerations. Specifically, patients with post-cardiac arrest [13], convulsive seizures [7], ICU readmissions, delayed initiation of mechanical ventilation (>24 hours), unknown transfer status, unknown sedation dosage, concurrent use of remifentanil and fentanyl, and tracheostomy at ICU admission were excluded to reduce clinical heterogeneity.

Exposure and outcomes

Patients were categorized into remifentanil (R group) and fentanyl (F group) groups. Pain was assessed using the behavioral pain scale (BPS) [14]; scores below four were targeted. Sedation depth was monitored with the Richmond Agitation-Sedation Scale [15]; scores between -2 and zero were targeted. Both pain and sedation were routinely assessed three times per day by the ICU nurses, respectively. In addition, assessments were performed whenever changes in the patient’s clinical condition were observed.

In the R group, administration was generally guided by an institutional protocol, which specified a starting dose of 0.025 μg/kg/minute and dose adjustments of ±0.025 μg/kg/minute based on BPS scores (e.g., increase if BPS > 4, decrease if BPS < 4). In contrast, fentanyl administration was at the discretion of the attending physician without a unified protocol. For both groups, sedatives (e.g., propofol, dexmedetomidine, midazolam) were adjusted based on clinical judgment and Richmond Agitation-Sedation Scale (RASS) monitoring. Medication dosage and assessment frequency were ultimately at the discretion of the attending physician according to clinical response, and no strict, unified protocol was applied throughout the entire study period. The primary outcome was mechanical ventilation duration. Secondary outcomes included the incidence of delirium, length of ICU stay, mortality at ICU discharge, and daily cost of analgesics or combined analgesic-sedative therapy. Other sedatives administered during mechanical ventilation included propofol, dexmedetomidine, and midazolam. These were selected and titrated at the discretion of the attending physician based on clinical need and sedation goals. We recorded the daily doses of each sedative to compare usage patterns between the groups.

Delirium was defined as a positive Confusion Assessment Method for the Intensive Care Unit (CAM-ICU) assessment [16] at any time during mechanical ventilation. Assessments were conducted three times daily by trained ICU nurses who had received standardized instruction in the use of the CAM-ICU. In this study's protocol, all patients were already receiving either remifentanil or fentanyl at the time of their first CAM-ICU assessment. Consequently, a formal pre-exposure baseline was not performed. Our analysis was therefore focused on comparing delirium incidence during the on-treatment period for these two opioids.

Analgesic costs were calculated based on remifentanil (for intravenous injection “Daiichi Sankyo”, ¥2118 per 5000 mcg) and fentanyl (for intravenous injection “Terumo”, ¥887 per 500 mcg), with currency conversion at ¥156.4 per USD as of December 31, 2024. Sedative costs during mechanical ventilation included dexmedetomidine, midazolam, propofol, ketamine, diazepam, and haloperidol.

Variables

We recorded demographics (age at admission, sex (male), and body mass index), clinical conditions (acute kidney injury within 24 hours), comorbidities (acquired immune deficiency syndrome, acute myeloid leukemia, multiple myeloma, lymphoma, maintenance dialysis, liver cirrhosis, liver failure, metastatic cancer, respiratory failure, heart failure, and immunosuppression); and severity scores (Acute Physiology and Chronic Health Evaluation (APACHE) II and III, Simplified Acute Physiology Score (SAPS II), and Sequential Organ Failure Assessment (SOFA)). Others were ICU interventions (use of intra-aortic balloon pump, non-invasive positive pressure ventilation, percutaneous cardiopulmonary support, veno-venous extracorporeal membrane oxygenation, continuous and intermittent renal replacement therapy, tracheostomy, plasma exchange, central venous line, arterial pressure line, and polymyxin B-immobilized fiber column.

Statistical analysis

The pooled standard deviation for the fentanyl and remifentanil groups was reported as 149.93, with a mean difference of -53.5 hours [17]. We set the type I error at 0.05 and the type II error at 0.2 (80% power) to calculate the required minimum sample size as 125 patients per group (total 250). A propensity score analysis was employed to address baseline differences between the R and F groups. Logistic regression models were used to calculate propensity scores, incorporating variables such as demographic characteristics, ICU admission comorbidities, severity scores, and ICU interventions. The discriminatory ability of the logistic regression model to differentiate patients in the R and F groups was evaluated using C-statistics. A C-statistic value between 0.6 and 0.9 was considered optimal for achieving sufficient discrimination without overfitting or insufficient separation between treatment groups [18]. Propensity score matching used one-to-one nearest-neighbor matching without replacement and a caliper width of 20% of the standard deviation of logit-transformed scores. Balance between groups was assessed using absolute standardized differences, with values <10% indicating balance. Patients with missing data were excluded from the analysis. To evaluate the robustness of our findings in light of the relatively small sample size, a sensitivity analysis was performed in the full cohort of patients before propensity score matching. Categorical variables are reported as numbers and percentages, and continuous variables as medians with interquartile range (IQR). Between-group comparisons of continuous variables, including the duration of mechanical ventilation, were conducted using the Mann-Whitney U test after matching. Risk differences and 95% confidence intervals (CIs) were calculated where appropriate. The median and IQR were calculated for hospital and ICU stay length. The Wilcoxon rank-sum test was used to compare the two groups. A two-sided P-value < 0.05 indicated significance. Statistical analyses were conducted using SPSS (version 29.0; IBM SPSS Statistics for Windows, IBM Corp., Armonk, NY) and R v.4.0.2 (R Foundation for Statistical Computing, Vienna, Austria).

## Results

Participant selection

**Figure 1:**
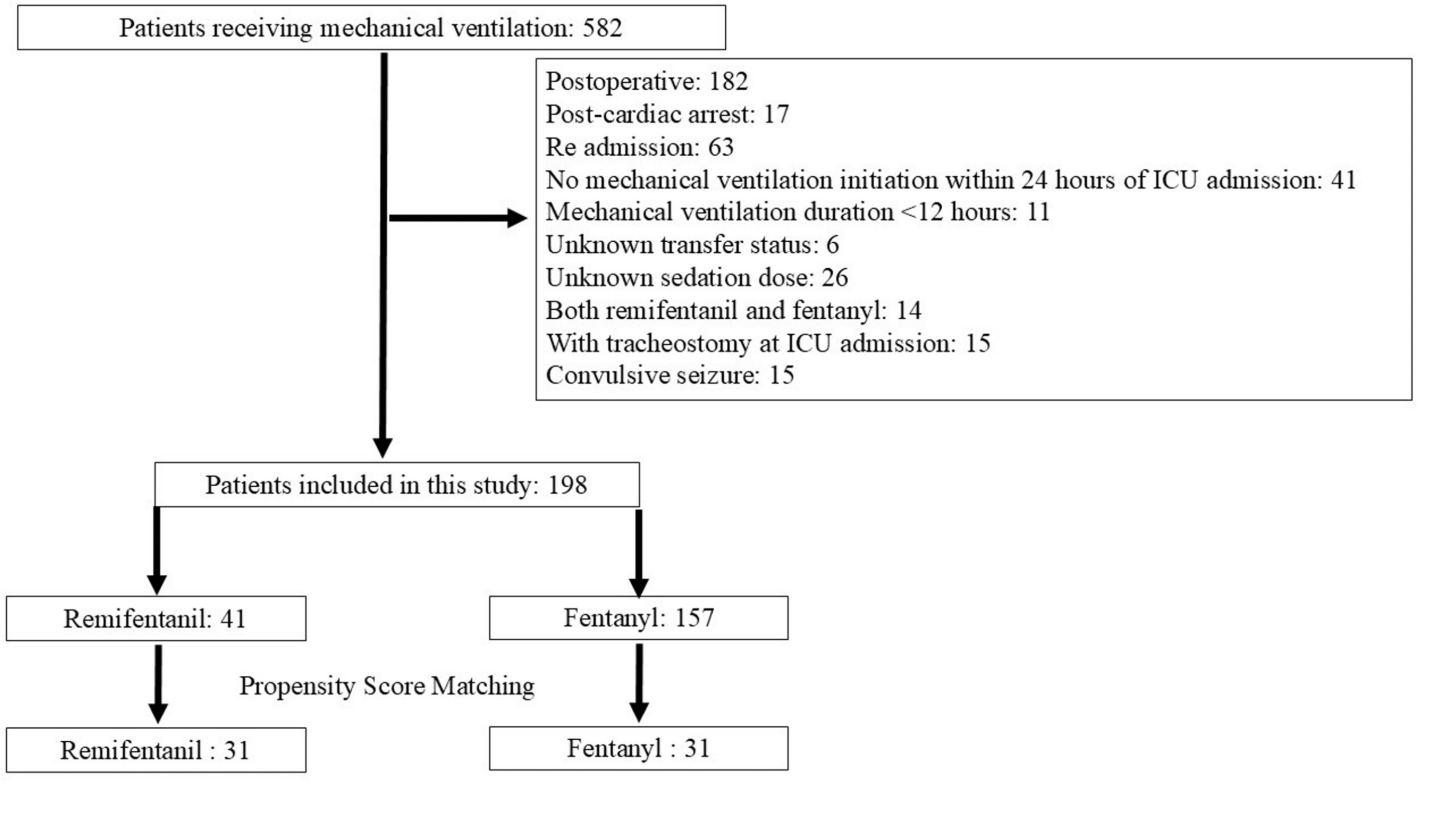
Patient selection flowchart Of the 582 patients who underwent mechanical ventilation, 198 met the inclusion criteria and were included in the study. Among these, 41 patients are assigned to the remifentanil group and 157 to the fentanyl group. After applying 1:1 propensity score matching, 31 patients were included in each group for comparative analysis.

Among the 582 patients who received mechanical ventilation during the study period, 384 were excluded based on the predefined criteria. Consequently, 198 patients met the inclusion criteria and were included in the initial analysis. Of these, 41 (20.7%) and 157 (79.3%) were assigned to the R and F groups, respectively (Figure 1).

Following propensity score-matching, 31 patients were included in each group for comparative analysis.

Participant characteristics

Baseline characteristics before propensity score matching demonstrated several imbalances between groups (Table 1).

**Table 1:** Characteristics of patients before propensity score matching AIDS: acquired immunodeficiency syndrome, AKI: acute kidney injury, AML: acute myeloid leukemia, APACHE II: Acute Physiology and Chronic Health Evaluation II, APACHE III: Acute Physiology and Chronic Health Evaluation III, BMI: body mass index, IABP: intra-aortic balloon pumping, NPPV: noninvasive positive-pressure ventilation, PCPS: percutaneous cardiopulmonary support, PMX: polymyxin B hemoperfusion, RRT: renal replacement therapy, SAPS II: Simplified Acute Physiology Score II, SMD: standardized mean difference, SOFA: Sequential Organ Failure Assessment, VV-ECMO: veno-venous extracorporeal membrane oxygenation

Variable	Remifentanil (N = 41)	Fentanyl (N = 157)	SMD
Age on Admission	69.0 (58.0, 79.0)	70.0 (58.0, 78.0)	0.11
Gender (Male)	27 (65.9)	116 (73.9)	0.18
BMI	23.8 (21.0, 25.5)	22.8 (20.1, 25.8)	0.17
AKI Within 24 hours	7 (17.1)	3 (1.9)	0.54
AIDS	0 (0)	0 (0)	<0.001
AML/MM	4 (9.8)	6 (3.8)	0.24
Lymphoma	2 (4.9)	3 (1.9)	0.16
Maintenance Dialysis	0 (0)	6 (3.8)	0.28
Liver Cirrhosis	5 (12.2)	7 (4.5)	0.28
Liver Failure	2 (4.9)	4 (2.5)	0.12
Cancer Metastasis	1 (2.4)	7 (4.5)	0.11
Respiratory Failure	3 (8.3)	8 (5.1)	0.092
Heart Failure	0 (0)	1 (0.6)	0.11
Immunosuppression	4 (9.8)	9 (6.3)	0.15
APACHE III Score	93.0 (61.0, 127.0)	92.0 (67.0, 117.0)	0.004
APACHE II Score	24.0 (19.0, 34.0)	24.0 (17.0, 30.0)	0.13
SAPS II Score	58.00 (35.0, 72.0)	56.0 (43.0, 71.0)	0.066
SOFA Score	11.0 (8.0, 13.0)	9.00 (7.0, 12.0)	0.18
IABP	0 (0)	0 (0)	<0.001
NPPV	5 (12.2)	6 (3.8)	0.31
PCPS	1 (2.4)	0 (0)	0.22
VV-ECMO	2 (4.9)	1 (0.6)	0.26
Continuous RRT	1 (2.4)	2 (1.3)	0.086
Intermittent RRT	5 (12.2)	27 (17.2)	0.14
Tracheostomy	6 (14.6)	31 (19.7)	0.14
Plasma Exchange	0 (0)	1 (0.6)	0.11
Central Venous Line	35 (85.4)	131 (83.4)	0.053
Arterial Pressure Line	41 (100.0)	157 (100.0)	<0.001
PMX	0 (0)	0 (0)	<0.001

The C-statistic calculated for propensity score matching was 0.78. After matching, most variables demonstrated standardized mean differences (SMDs) below the threshold of 0.1, indicating an appropriate balance between groups (Table 2).

**Table 2:** Characteristics of patients after propensity score matching AIDS: acquired immunodeficiency syndrome, AKI: acute kidney injury, AML: acute myeloid leukemia, APACHE II: Acute Physiology and Chronic Health Evaluation II, APACHE III: Acute Physiology and Chronic Health Evaluation III, BMI: body mass index, IABP: intra-aortic balloon pumping, NPPV: noninvasive positive-pressure ventilation, PCPS: percutaneous cardiopulmonary support, PMX: polymyxin B hemoperfusion, RRT: renal replacement therapy, SAPS II: Simplified Acute Physiology Score II, SMD: standardized mean difference, SOFA: Sequential Organ Failure Assessment, VV-ECMO: veno-venous extracorporeal membrane oxygenation

Variable	Remifentanil (N = 31)	Fentanyl (N = 31)	SMD
Age on Admission	69.0 (58.5, 79.5)	76.0 (68.5, 80.0)	0.37
Gender (Male)	22 (71.0)	22 (71.0)	<0.001
BMI	23.0 (21.0, 25.3)	23.9 (19.8, 27.2)	0.036
AKI Within 24 hours	1 (3.2)	2 (6.5)	0.15
AIDS	0 (0)	0 (0)	<0.001
AML/MM	3 (9.7)	2 (6.5)	0.12
Lymphoma	2 (6.5)	1 (3.2)	0.15
Maintenance Dialysis	0 (0)	0 (0)	<0.001
Liver Cirrhosis	2 (6.5)	4 (12.9)	0.22
Liver Failure	1 (3.2)	1 (3.2)	<0.001
Cancer Metastasis	1 (3.2)	1 (3.2)	<0.001
Respiratory Failure	3 (9.7)	4 (12.9)	0.10
Heart Failure	0 (0)	0 (0)	<0.001
Immunosuppression	2 (6.5)	2 (6.5)	<0.001
APACHE III Score	80.0 (59.0, 112.5)	85.0 (69.0, 113.5)	0.11
APACHE II Score	23.0 (18.5, 28.0)	24.0 (18.5, 29.0)	0.077
SAPS II Score	54.0 (34.5, 70.0)	52.0 (45.0, 71.5)	0.20
SOFA Score	9.0 (6.5, 12.0)	10.0 (7.0, 13.0)	0.12
IABP	0 (0)	0 (0)	<0.001
NPPV	3 (9.7)	2 (6.5)	0.119
PCPS	0 (0)	0 (0)	<0.001
VV-ECMO	1 (3.2)	1 (3.2)	<0.001
Continuous RRT	0 (0)	0 (0)	<0.001
Intermittent RRT	3 (9.7)	4 (12.9)	0.102
Tracheostomy	5 (16.1)	9 (29.0)	0.312
Plasma Exchange	0 (0)	0 (0)	<0.001
Central Venous Line	25 (80.6)	26 (83.9)	0.085
Arterial Pressure Line	31 (100.0)	31 (100.0)	<0.001
PMX	0 (0)	0 (0)	<0.001

The median (IQR) analgesic dosage during mechanical ventilation was 0.12 (0.07-0.20) and 1.10 (0.32-3.5) mcg/kg/hour in the R and F groups, respectively.

Clinical outcomes

**Figure 2:**
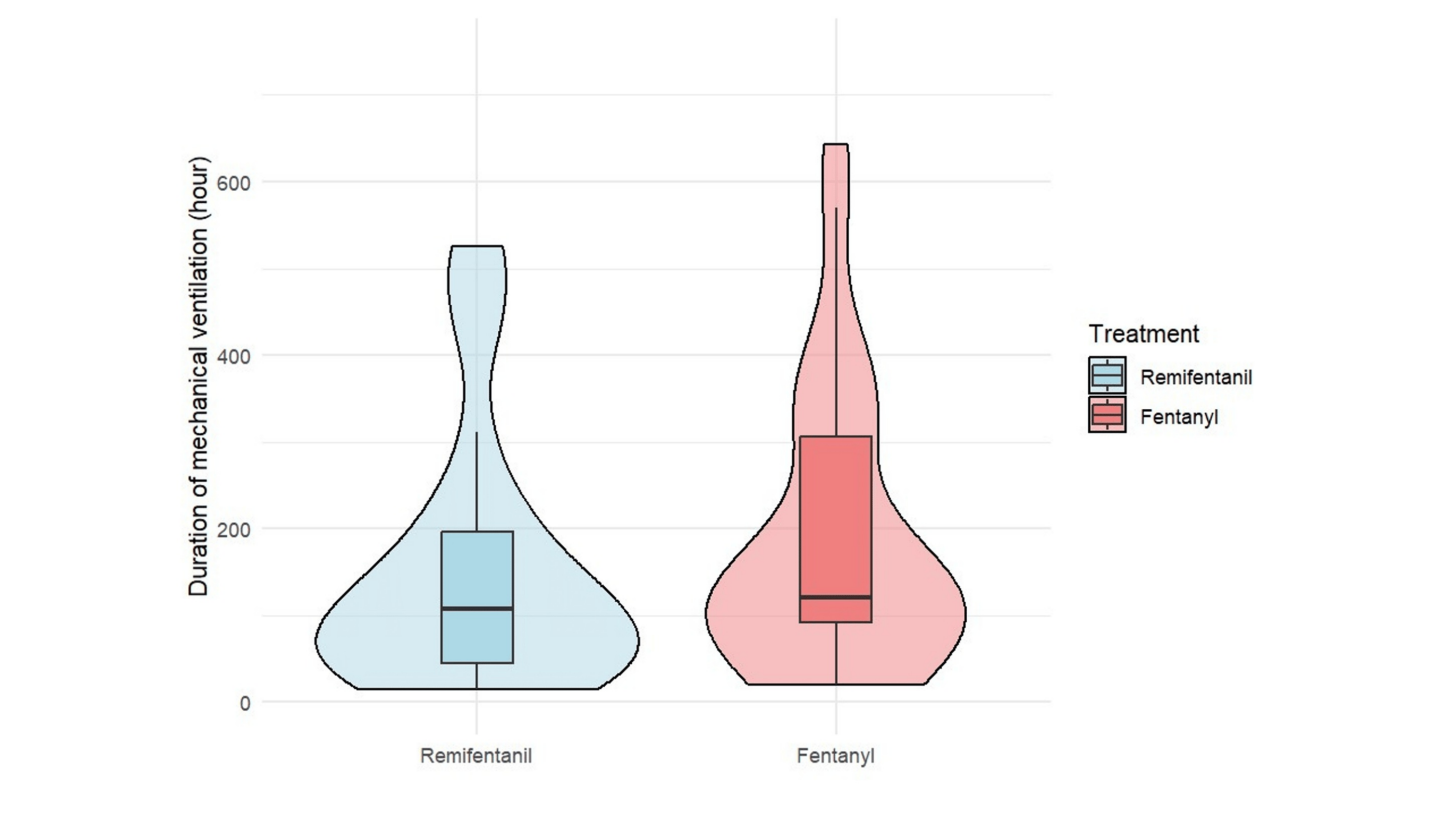
Comparison of mechanical ventilation duration (hours) between remifentanil and fentanyl groups The boxes represent the interquartile range (IQR), and the horizontal lines within the boxes indicate median values. The median duration of mechanical ventilation was 107.5 hours in the remifentanil group and 124.1 hours in the fentanyl group. The median difference (remifentanil-fentanyl) was -16.6 hours (95% CI: -147.0 to 26.2). The comparison was performed using the Mann-Whitney U test (P = 0.15).

The median (IQR) duration of mechanical ventilation was 107.5 hours (45.6-196.8) and 124.1 hours (93.8-324.0) in the R and F groups, respectively, with a median difference of -16.6 hours (95% CI: -147.0-26.2; P = 0.15) (Table 3; Figure 2).

**Table 3:** Outcomes after propensity score matching Values are presented as numbers (%) or medians (Q1, Q3).

Outcome	Remifentanil (N = 31)	Fentanyl (N = 31)	Median Difference (95% CI)	P-value
Primary outcome				
Duration of mechanical ventilation (hour)	107.5 (45.6-196.8)	124.1 (93.8-324.0)	-16.6 (-147.0 to 26.2)	0.15
Secondary outcomes				
Delirium incidence (%)	77.4% (N = 31)	96.4% (N = 28)	N/A	0.03
Length of ICU stay (days)	7.8 (4.2-8.5)	7.8 (5.9-15.9)	-0.01 (-8.7 to 1.6)	0.24
Mortality at ICU discharge	9.7%	12.9%	-	0.69
Daily analgesics costs ($)	29.5 (25.0-34.3)	13.9 (11.5-16.7)	15.6 (9.5 to 21.7)	<0.01
Daily sedatives costs ($)	16.9 (11.3-25.0)	12.8 (16.8-35.1)	4.17 (-18.6 to 13.0)	0.86
Daily analgesics and sedatives costs ($)	45.3 (40.1-51.2)	26.2 (20.1-52.5)	19.16 (-10.1 to 29.6)	0.13

**Figure 3:**
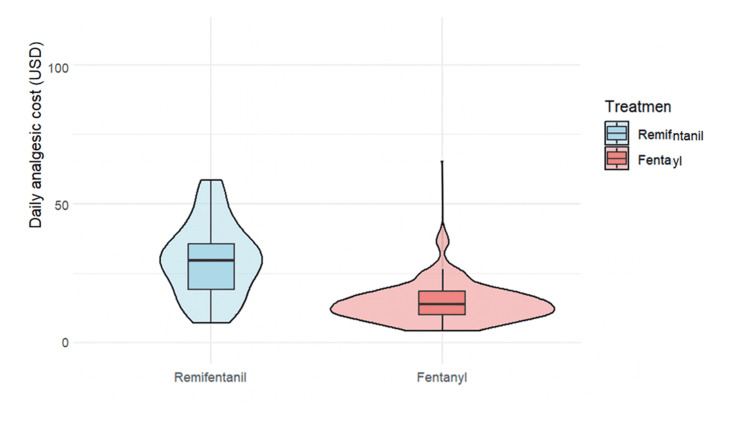
Daily analgesic and sedative costs (in USD) between remifentanil and fentanyl groups Daily analgesic and sedative costs (in USD) for the remifentanil (R) and fentanyl (F) groups are presented. The median cost was $45.3 in the R group and $26.2 in the F group. No significant difference was observed between the two groups (Mann-Whitney U test, P = 0.13).

**Figure 4:**
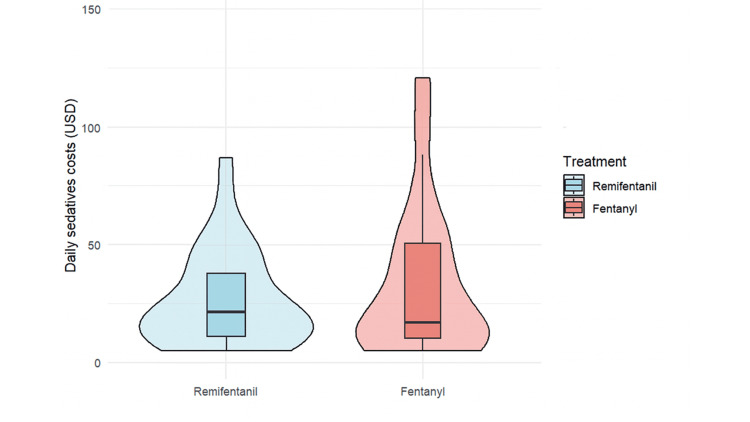
Daily sedative costs (in USD) between remifentanil and fentanyl groups Daily sedative costs (in USD) for the remifentanil (R) and fentanyl (F) groups are presented. The median cost was significantly higher in the R group ($16.9) than in the F group ($12.8), with a significant difference (Mann-Whitney U test, P = 0.86).

**Figure 5:**
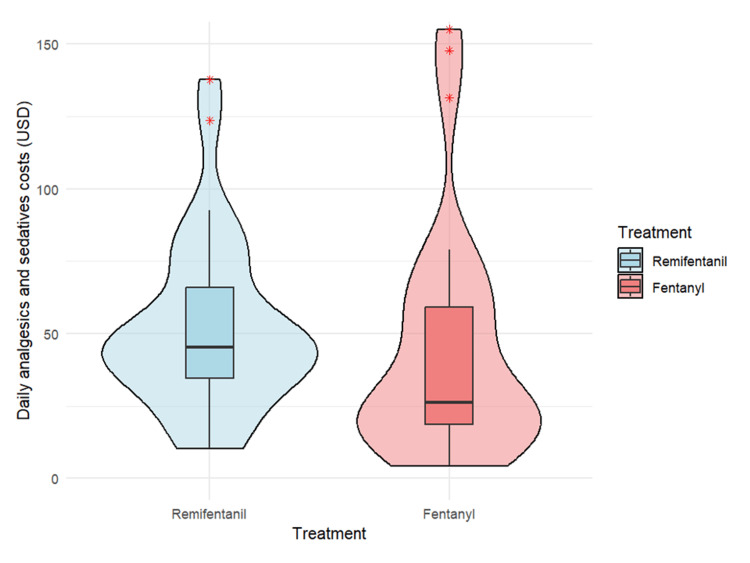
Daily analgesic costs (in USD) between remifentanil and fentanyl groups Daily analgesic costs (in USD) for the remifentanil (R) and fentanyl (F) groups are presented. The median cost was significantly higher in the R group ($29.5) than in the F group ($13.9), with a significant difference (Mann-Whitney U test, P < 0.01).

As a sensitivity analysis, we also compared the duration of mechanical ventilation in the full, unmatched cohort. The median (IQR) ventilation duration was 107.5 hours in the R group and 115.0 hours in the F group. The difference was not statistically significant (P = 0.38, Mann-Whitney U test). Analysis of secondary outcomes revealed that delirium incidence was significantly lower in the R group (77.4% vs. 96.4%; χ²(1) = 4.99, P = 0.03, φ = 0.29) (Table 3). The median ICU stay was comparable between groups at 7.8 days in both groups (P = 0.24) (Table 3). Although daily analgesic costs were significantly higher in the R group ($29.5 vs. $13.9; P < 0.01), daily sedative costs were similar between groups ($16.9 vs. $12.8; P = 0.86) (Table 3; Figures 3-5).

The total daily analgesic and sedative costs were similar between groups ($45.3 vs. $26.2; P = 0.13). There were no significant differences between the groups in the daily doses of dexmedetomidine, midazolam, or propofol administered during mechanical ventilation (Table 4).

**Table 4:** Daily doses of sedative agents during mechanical ventilation in the remifentanil and fentanyl groups Values are presented as numbers (%) or medians (Q1, Q3).

Outcome	Remifentanil (N = 31)	Fentanyl (N = 31)	P-value
Sedation drugs			
Dexmedetomidine (μg/day)	89.3 (0.0-197.2)	60.7 (4.0-176.7)	0.96
Propofol (mg/day)	368.7 (52.5-1728.6)	577.9 (145.5-2076.7)	0.43
Midazolam (mg/day)	7.8 (1.4-18.1)	4.7 (0.0-16.1)	0.42

## Discussion

Summary of findings

This retrospective study compared remifentanil and fentanyl in critically ill patients on mechanical ventilation. Ventilation duration and ICU stay were similar, but delirium incidence was significantly lower in the R group. Although analgesic costs were higher, total sedation and analgesia costs showed no difference. These findings suggest potential benefits of remifentanil in ICU sedation management.

Mechanistic rationale

Remifentanil possesses ultra-short-acting properties and is metabolized by nonspecific esterases, making its pharmacokinetics independent of liver or kidney function. This allows for rapid titration and minimal drug accumulation even in patients with organ dysfunction - a common condition in the ICU [19]. These features enable earlier awakening and more consistent neurological assessments, including delirium monitoring [10]. In contrast, fentanyl is primarily metabolized by the liver and may accumulate in cases of organ dysfunction [6,7], potentially leading to delayed awakening and greater difficulty in delirium assessment. These pharmacological differences may partly explain the observed reduction in delirium incidence in the R group.

Mechanical ventilation duration

In our study, no reduction in mechanical ventilation duration was observed. This result aligns with the findings of a network meta-analysis comparing remifentanil and fentanyl in ICU patients [20]. However, this meta-analysis did not discuss Rajamani et al.’s study [11], and therefore, cannot fully represent outcomes in critically ill patients. The effect of remifentanil on reducing mechanical ventilation duration in this population appears limited, warranting further investigation. Two key randomized controlled trials (RCTs) have been reported that primarily focused on comparing remifentanil and fentanyl in critically ill medical patients.

Rajamani et al. conducted a single-center RCT [11] including 208 patients, with 77% in the R group and 71% in the F group being medical ICU patients, whereas 20% were postoperative patients. Remifentanil significantly reduced mechanical ventilation duration compared to fentanyl (median: 2.2 days vs. 3.1 days, P = 0.001). However, 19% of the patients in that study received remifentanil and fentanyl, making it difficult to interpret the results as a direct comparison of monotherapies.

Breen et al. [17] conducted a multicenter RCT including 105 patients across 15 centers. The remifentanil-based regimen significantly reduced mechanical ventilation duration (median: 94.0 hours vs. 147.5 hours, P = 0.03). However, the inclusion of morphine in the control group may have influenced the outcomes, potentially accounting for the observed differences in ventilation duration. Although both studies demonstrated a significant reduction in mechanical ventilation duration with remifentanil-based regimens, they differed in design and limitations.

In our study, the R group showed a trend toward reducing mechanical ventilation duration by approximately 17 hours compared to the F group, but this did not reach significance. The lack of statistical power in our analysis may explain the absence of a significant difference. Additionally, the differences in patient populations, including the exclusive use of single agents in our study, and the design variations across the studies could contribute to the discrepancy in the findings.

Delirium

Our results suggest that the use of remifentanil, compared to fentanyl, may reduce delirium in critically ill patients. These findings are consistent with previous results.

In Rajamani et al.’s study [11], remifentanil significantly reduced delirium incidence compared to fentanyl in critically ill medical patients (15/105 vs. 28/127, P = 0.01). Similarly, Liu et al. [9], who focused on postoperative surgery patients, reported a lower delirium incidence following remifentanil administration compared to fentanyl administration (8/35 vs. 14/35, P = 0.02). This protective effect may be attributable to remifentanil’s unique pharmacokinetic properties. Its ultra-short-acting profile, rapid titratability, and metabolism by nonspecific esterases (independent of liver and kidney function) enable more precise sedation control, reducing drug accumulation and facilitating earlier awakening [10]. These characteristics may allow for more consistent and timely delirium assessments, contributing to reduced delirium incidence. Additionally, recent insights into the relationship between pain and delirium indicate that the risk factor for delirium is not the presence of pain but rather inadequately managed pain [21]. The rapid analgesic effects of remifentanil allow for effective pain control, reducing stress and discomfort, which may further contribute to its ability to mitigate delirium [22]. In the R group, slightly higher doses of midazolam and dexmedetomidine were used. This may reflect the clinical practice in Japan, where remifentanil bolus administration is not permitted. As such, clinicians may have supplemented with bolus-available agents like midazolam or with continuous agents like dexmedetomidine to enhance sedation. Despite the increased use of these adjunctive sedatives, the remifentanil group demonstrated improved outcomes in terms of delirium incidence, highlighting the potential clinical advantages of remifentanil-based sedation strategies.

Cost considerations for remifentanil in the ICU

Evaluating the costs of remifentanil in the ICU requires analyzing direct drug costs and potential cost savings from improved clinical outcomes. Although remifentanil is generally more expensive than traditional analgesics and sedatives, such as fentanyl and midazolam, its clinical benefits may offset these higher acquisition costs. Al et al. [23] reported that remifentanil could reduce mechanical ventilation duration, ICU stay length, and overall hospital stay length, ultimately lowering healthcare resource utilization and total costs. In this study, a direct comparison of remifentanil and fentanyl drug costs confirmed that remifentanil was more expensive.

When combined costs for analgesics and sedatives were considered, the difference was not significant. This may be explained by variability in sedative use in the fentanyl group. While the remifentanil group showed higher analgesic costs, sedative use - especially agents like propofol and dexmedetomidine - was more variable and, in some cases, more intensive in the fentanyl group. This variability may have offset the cost difference, resulting in no statistically significant difference in total daily medication costs between the two groups.

Although non-drug-related costs could not be calculated, our findings, combined with previous results [11], suggest that remifentanil may help reduce adverse events, such as delirium. Delirium in the ICU has been linked to long-term cognitive impairment, which can negatively impact patient outcomes and markedly increase healthcare costs [24]. A previous study [25] suggested that sedation strategies using fentanyl or midazolam are associated with a higher incidence of delirium, which may lead to prolonged ICU stay and increased healthcare costs. While the exact economic burden of fentanyl-induced delirium was not quantified, these findings emphasize the potential economic benefits of delirium-sparing sedation strategies such as those involving remifentanil.

Limitations

This study has some limitations. First, as a single-center, retrospective observational study, the findings may lack generalizability to other healthcare settings or institutions. The study design inherently limits the ability to establish causation; therefore, the results should be interpreted cautiously.

Second, although propensity score matching was employed to minimize baseline differences between groups, the influence of unmeasured confounding factors cannot be entirely ruled out. In particular, baseline cognitive function was not available in the dataset, and pre-existing cognitive impairment or undiagnosed neurodegenerative conditions, known risk factors for ICU delirium, could not be accounted for, potentially biasing the analysis of delirium incidence.

Third, the relatively small sample size, particularly in the matched cohorts, may have reduced the statistical power to detect differences in specific outcomes, such as the duration of mechanical ventilation, ICU stay, and associated costs. In particular, while the median difference in mechanical ventilation duration appeared clinically relevant, the wide 95% CI (-147.0 to 26.2 hours) and non-significant P-value (P = 0.15) suggest substantial imprecision. This underscores the limited power of the study and highlights the need for further research with larger sample sizes.

Fourth, the cost analysis focused exclusively on medication expenses incurred during the mechanical ventilation period and did not consider other potentially significant cost components, such as personnel, equipment, ICU stay duration, infection management, or treatment of complications like pneumonia, nor post-ICU care. This limited scope may underestimate the true economic impact of the sedation strategy.

Fifth, although we applied propensity score matching to adjust for observed baseline characteristics, unmeasured confounding factors, such as physicians’ subjective decisions based on anticipated weaning success or clinical trajectory, may have influenced the choice of remifentanil versus fentanyl. These clinical judgments were not captured in the dataset and could not be included in the propensity score model. These may include variations in bedside nursing practices related to sedation titration, differences in ICU protocols for weaning from mechanical ventilation, or unrecognized neurological or psychiatric conditions not captured by standard severity scores. Such factors could have influenced both the choice of sedative and the observed outcomes, including delirium incidence and duration of mechanical ventilation.

Sixth, the lack of a standardized sedation protocol across all patients may have introduced treatment variability, particularly in sedative choice and titration practices. This heterogeneity could have affected key outcomes such as delirium incidence or duration of mechanical ventilation, and may represent an additional source of confounding.

Seventh, the use of additional sedatives such as propofol, dexmedetomidine, and midazolam may have influenced outcomes like mechanical ventilation duration or delirium incidence. However, we observed no significant differences in daily sedative doses between the remifentanil and fentanyl groups (Table 4), which may mitigate confounding due to differential sedative exposure. Lastly, the findings regarding cost-effectiveness may not apply to other regions or healthcare systems with differing economic structures or medication pricing. Additionally, complications associated with remifentanil use, such as bradycardia and hypotension, were considered for investigation, but data on these complications were not collected.

## Conclusions

This retrospective study suggests that remifentanil may be associated with a lower incidence of delirium compared to fentanyl in mechanically ventilated ICU patients, with similar durations of ventilation and ICU stay. Although remifentanil was associated with higher analgesic costs, total sedation and analgesia costs did not differ significantly between groups. Given the retrospective design, single-center setting, and limited sample size, the findings should be interpreted cautiously. Despite the use of propensity score matching, unmeasured confounding remains a potential source of bias. Future prospective studies with larger cohorts are warranted to validate these findings and comprehensively assess the clinical and economic impact of remifentanil in ICU sedation strategies.
